# Severe Cholestasis and Bile Cast Nephropathy Induced by Anabolic Steroids Successfully Treated with Plasma Exchange

**DOI:** 10.1155/2017/4296474

**Published:** 2017-12-17

**Authors:** Christelle El Khoury, Toni Sabbouh, Hussein Farhat, Antoine Ferzli

**Affiliations:** ^1^Family Medicine Division, Lebanese American University Medical Center, Beirut, Lebanon; ^2^Internal Medicine Division, Morristown Medical Center, Morristown, NJ, USA; ^3^Laboratory Medicine Division, Lebanese American University Medical Center, Beirut, Lebanon; ^4^Gastroenterology Division, Lebanese American University Medical Center, Beirut, Lebanon

## Abstract

We report a case of a bodybuilder who took a regimen of anabolic steroids containing stanozolol and testosterone propionate for 8 weeks which led to the development of jaundice and severe pruritus with serum total bilirubin reaching 41.22 mg/dL. Despite supportive care with fluid and albumin therapy, serum creatinine was progressively increasing. He underwent 6 successful sessions of plasma exchange (PE) with marked improvement at the end of the sessions. Three months after discharge, the patient's creatinine and total bilirubin levels were 1.08 mg/dL and 1.2 mg/dL, respectively.

## 1. Introduction

Anabolic androgenic steroid abuse and misuse is common not only among athletes but also among the general population affected by body image disturbance or eating disorders [1, 2]. Stanozolol and testosterone propionate are part of the 17 alpha-alkylated (17aa) steroids that have been associated with cholestatic jaundice usually resolving after drug discontinuation [3].

Although the effects of these drugs are reversible on the liver, they have some serious side effects related to renal failure, adrenal changes, infections, and malnutrition [4, 5]. A review done by Modlinski et al. highlighted several cases in which anabolic steroids were the culprits of severe rare conditions including Wilms tumor and hepatic tumors requiring liver transplantation [4, 6]. We report a case of cholestatic jaundice and acute kidney injury in a bodybuilder using stanozolol.

## 2. Case Report

A 35-year-old male was admitted to the hospital for progressive jaundice, colicky abdominal pain, vomiting, and severe pruritus. He also reported dark urine and pale stools. He had no significant past medical history except for dyslipidemia on lifestyle modification and binge drinking of approximately 1 liter during the weekends. Twelve weeks prior to presentation, he started a regimen for weight loss and muscle building consisting of 3 different types of anabolic steroids: stanozolol (100 mg, 3 times/week, intramuscular), trenbolone (100 mg, 3 times/week, intramuscular), and testosterone propionate (50 mg, 3 times/week, intramuscular). In addition to the anabolic steroids, he was taking 12 capsules daily consisting of dietary supplements such as T3 liothyronine, creatine, conjugated linoleic acid with olive oil, and amino acids. Five weeks prior to presentation, he reported yellowish skin discoloration to his local physician. His laboratory tests were significant for disturbed liver function tests: total bilirubin 8.4 mg/dL (0–0.2 mg/dL), direct bilirubin 6.13 mg/dL (0-1 mg/dL), aspartate aminotransferase 139 U/L (<41 U/L), alanine aminotransferase 450 U/L (<42 U/L), alkaline phosphatase 163 U/L (40–130 U/L), and gamma-glutamyl transpeptidase (GGT) 187 mg/dL (<71 mg/dL). Abdominal ultrasound was done and showed a normal liver. After ruling out viral (Epstein–Barr virus (EBV), cytomegalovirus (CMV), hepatitis A virus (HAV), hepatitis B virus (HBV), hepatitis C virus (HCV), and varicella) and autoimmune (negative for anti-smooth muscle antibody, anti-liver kidney microsomal antibody (anti-LKM), anti-mitochondrial antibody (AMA), and anti-neutrophil cytoplasmic antibody (ANCA)) causes of acute hepatitis, drug-induced liver injury was suspected, and he was advised to stop the anabolic steroids.

However, the patient reported worsening of his symptoms which were nausea, vomiting, severe itching, and colicky abdominal pain requiring admission to the hospital. On the first day of hospitalization, the patient was alert, oriented, and afebrile, with a blood pressure of 121/71 mmHg, and pulse oximetry reading showed SpO_2_ of 98% on room air. His physical examination was significant for scleral icterus, jaundice, and scratches on bilateral extremities due to severe pruritus. The abdomen was soft and nondistended, and there was no hepatomegaly. Laboratory data showed a hemoglobin level of 13.5 g/dL and elevated serum creatinine of 2.2 mg/dL, and abnormal liver function tests are now in favor of a cholestatic pattern: total bilirubin 37.9 mg/dL (0–0.2 mg/dL), direct bilirubin 32.1 mg/dL (0-1 mg/dL), indirect bilirubin 5.8 mg/dL (<1 mg/dL), aspartate aminotransferase 41 U/L (<41 U/L), alanine aminotransferase 33 U/L (<42 U/L), and alkaline phosphatase 329 U/L (40–130 U/L).

**Figure 1:**
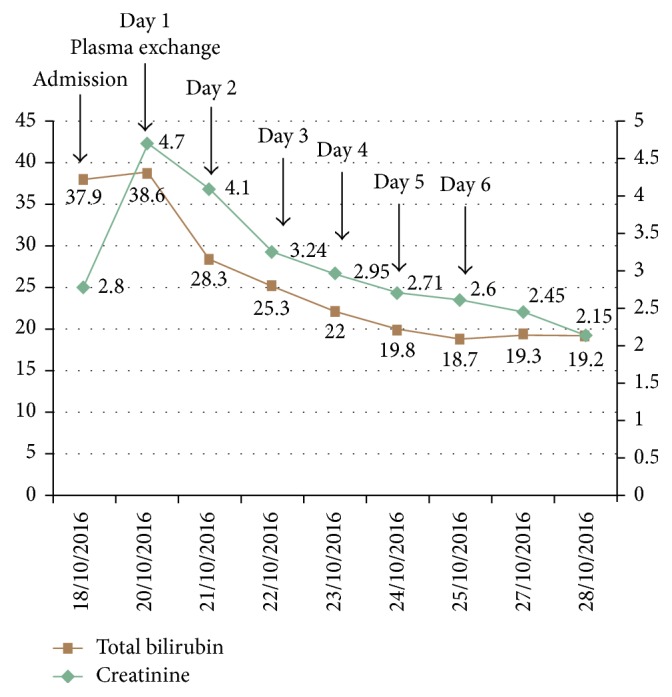
Trend of serum total bilirubin and creatinine levels during hospital stay.

Urine appeared icteric in color, with pH 8.5, bilirubin 3+, and no casts. Abdominal ultrasound showed homogeneous hepatosplenomegaly with normal echotexture and no biliary tree dilatation. The patient was diagnosed with liver injury resulting in prolonged cholestasis and acute kidney injury. Supportive care was provided with intravenous fluids mainly sodium bicarbonate to prevent cast formation, albumin, ursodeoxycholic acid (15 mg/kg), and hydroxyzine for pruritus. Nephrology was consulted and it supported the diagnosis of bile cast nephropathy due to the prolonged exposure to high levels of bilirubin and increase in creatinine. However, bile cast nephropathy could not be confirmed by renal biopsy due to patient refusal. The patient's status was getting worse so the decision was to initiate urgent plasma exchange on day 3 of admission and to replace it with donors' fresh frozen plasma. This decision was supported by the study by Keklik et al. who found that plasma exchange is efficient in lowering the levels of bilirubin [7]. The equipment used was Spectra Optia® Apheresis System by Terumo BCT, and citrate dextrose was used as the anticoagulant. The plasma volume used in each of the 6 sessions were 3800 ml, 3200 ml, 4000 ml, 3000 ml, 2800 ml, and 3400 ml, respectively, corresponding to a total of 20,200 ml. He tolerated all 6 sessions well and was discharged home with total bilirubin 19.2 mg/dL and serum creatinine 2.15 mg/dL as shown in Figure 1 and on ursodeoxycholic acid and antihistamines. A liver assessment scan (fibroscan) done during this period showed a fibrosis score of F3.

Three weeks after discharge, the patient was progressively showing clinical and laboratory improvement, and he was completely asymptomatic after three months with a creatinine level of 1.08 mg/dL and a total bilirubin level of 1.2 mg/dL.

Another fibroscan was repeated nine months after his discharge from the hospital showing minimal steatosis and no fibrosis F0 (CAP = 231 dB/m, IGR = 26, E = 4.2 kPa). The patient was doing well otherwise, except for excessive alcohol intake consisting of seven beers daily.

## 3. Discussion

Stanozolol and testosterone propionate, metabolized by the cytochrome P450 system [8], are part of the 17 alpha-alkylated (17aa) steroids that have been linked to liver damage such as cholestatic jaundice, peliosis hepatis, hepatic adenoma, nodular regeneration, and hepatocellular carcinoma [9–11]. It is not well understood how the C-17 substituted androgens cause cholestasis; it may be due to partial lack or variant of bile salt transporter proteins [3].

Cholestatic jaundice is related to dose and treatment duration and usually arises within 1 to 4 months of starting therapy and up to 6 to 24 months [12]. It is usually transient, and complete recovery after drug cessation may take several months [3]. The first reported case with stanozolol was in 1994, a 26-year-old athlete, in whom severe cholestasis and ATN (acute tubular necrosis) developed after he used five times the therapeutic dosage of stanozolol. He was treated supportively with normalization of his biliary enzymes 5 months after stanozolol was discontinued [13].

When the bilirubin level exceeds 20 mg/ml thus exceeding the albumin capacity for binding bilirubin, accumulation in the renal cells may occur causing kidney damage [14]. Bile cast nephropathy, also known as cholemic nephrosis, is a rare entity characterized by progressive renal impairment in patients with elevated serum bile salts and hyperbilirubinemia either by direct bile and bilirubin toxicity or tubular obstruction [15]. Reported cases about bile cast nephropathy due to cholestatic jaundice after using anabolic androgenic steroids include a bodybuilder abusing a regimen of steroids including stanozolol that were successfully treated with plasmapheresis [5] and 2 cases with stanozolol used for 2 months successfully treated with hemodialysis [16, 17].

Plasma exchange (PE) is a therapeutic procedure in which a large volume of the patient's plasma is removed and replaced with some form of replacement fluid, whereas plasmapheresis does not require plasma replacement since a smaller amount of plasma is removed [18, 19]. Additionally, PE is capable of removing noncellular blood constituents, including inflammatory markers and bilirubin, that are formed in liver injury [7, 20]. Although previous cases of bile cast nephropathy secondary to anabolic steroids were successfully managed by either plasmapheresis [5] or hemodialysis [16, 17], we decided to treat our patient's condition with PE supported by the findings published by Keklik et al. The patient's clinical status showed rapid improvement with PE; his pruritus decreased remarkably after each session, and his kidney and liver functions progressively normalized. We remained in close follow-up with the patient after he was discharged from the hospital, and his labs returned completely to baseline with a F0 score on fibroscan done after 9 months.

The aim of this case is to report a successful management of severe cholestasis and bile cast nephropathy secondary to anabolic steroids using plasma exchange. This case further adds on the serious side effects of these anabolic androgenic steroids among athletes and the need to increase awareness for this growing problem. Additionally, PE could also be considered in the management of pruritus in these patients. Finally, this report demonstrates how a liver stiffness assessment (fibroscan) is unreliable in patients with extrahepatic cholestasis showing falsely high values that may be related to increased intracellular pressure due to impaired bile flow, tissue swelling, inflammation, and edema [21].

The limitation of our case is that we were not able to perform a kidney biopsy to document bile casts and to rule out synergistic or concurrent hepatotoxicity from the other ingredients (i.e., trenbolone) in the regimen used due to patient refusal.
